# Classification of plastic surgery malpractice complaints brought before the São Paulo Medical Board that were treated as professional-misconduct cases: a cross-sectional study

**DOI:** 10.1590/1516-3180.2019.0363.09122019

**Published:** 2020-03-09

**Authors:** Paulo Cézar Mariani, Clóvis Francisco Constantino, Rui Nunes

**Affiliations:** I MD. Doctoral Student, Faculdade de Medicina da Universidade do Porto, Porto, Portugal.; II PhD. Physician and Professor, Universidade de Santo Amaro (UNISA), São Paulo (SP), Brazil.; III PhD. Professor, Faculdade de Medicina da Universidade do Porto, Porto, Portugal.

**Keywords:** Process assessments, health care, Medical errors, Surgery, plastic, Medical ethics, Medical board investigations, Classification of complaints

## Abstract

**BACKGROUND:**

Nowadays, there is an ethical and moral necessity to establish rules that govern professional attitudes and conduct. In the medical field, these rules are multifaceted, given the health consequences inherent to medical procedures. Ethics is an even more delicate subject when it comes to plastic surgery, since one of the aims of this particular medical specialty is esthetic improvement of the body.

**OBJECTIVE:**

To survey and classify São Paulo State Medical Board investigations of plastic-surgery complaints that were treated as professional-misconduct cases between 2007 and 2016.

**DESIGN AND SETTING:**

Cross-sectional study conducted in a medical council.

**METHODS:**

A total of 360 cases were reviewed. Among these, 8 (2.23%) were dismissed, 1 (0.27%) became an administrative lawsuit and 351 (97.50%) were treated as professional-misconduct cases.

**RESULTS:**

A breakdown of the complaints filed over the nine-year period showed that complaints concerning malpractice were the most common (28.43%), followed by those regarding medical advertising (24.19%) and poor doctor-patient relationships (10.39%).

**CONCLUSION:**

Overall, the number of complaints lodged decreased over the last two years reviewed, although complaints regarding malpractice and poor doctor-patient relationships increased by 10% over the same period. In order to further reduce the number of medical board investigations, the medical establishment needs to carefully review the medical training of students and doctors at every stage of their careers.

## INTRODUCTION

Regional Medical Councils (Conselhos Regionais de Medicina, CRMs) monitor and ensure proper ethical conduct of doctors throughout Brazil. They are entrusted with encouraging upright practice and championing the professional prestige and regard of the medical profession as a whole and of all who legally practice medicine.^1^ CRMs also have the mission of championing the independence of and free legal practice of medicine and defending doctors’ rights, while respecting the principles and guidelines contained in the Code of Medical Ethics and the resolutions of the Federal Medical Council (Conselho Federal de Medicina, CFM).

They issue medical documentation and assess working conditions. Furthermore, they review, investigate and decide on the licensing status of doctors who breach professional rules and standards. Board oversight extends from individual activity to both public and private institutional operations, including the entire medical hierarchy of institutions that directly or indirectly provides healthcare services. This means that the CRMs have the power to authorize, order partial suspense of or prohibit the exercising of any activities, along with inspection of services and activities pursued by individuals or institutions in accordance with the law.^2^

Since the CFM is a federally mandated autonomous agency, the CRMs are authorized to discipline medical activity via resolutions that determine medical permissions and prohibitions, and to investigate complaints and determine applicable disciplinary sanctions when the Code of Medical Ethics has been violated. Therefore, the CRMs have the legal prerogative to accept complaints, investigate the facts, judge the doctors involved and weigh up which sanctions are to be applied to each type of violation.

The numbers of formal complaints against doctors’ attitudes that have resulted in investigations have been growing both domestically and internationally.^3^ This has been seen especially within the civil courts, which are concerned with damages, and within the administrative courts, which are concerned with medical board investigations and reviews.

In 2017, the Courts of Justice of the State of Pará (Tribunal de Justiça do Pará, TJPA) reviewed criminal cases under the search term “medical malpractice.” Cases were assigned to medical specialties as follows: eight cases in obstetrics/gynecology; four in emergency care; two in general surgery; one in anesthesiology/plastic surgery; one in ophthalmology; one in orthopedics; and one in radiology. The courts concluded that surgery and emergency medicine, primarily obstetrics/gynecology, were the medical specialties against which most complaints and lawsuits had been filed.^4^

Once a complaint has been lodged, the full regional medical board or the board’s investigation committee opens an investigation to assess the facts of the case.

In the state of São Paulo, by law, the Regional Medical Council of the State of São Paulo (Conselho Regional de Medicina do Estado de São Paulo, CREMESP) must initially accept any complaint lodged by any citizen against doctors who practice within its jurisdiction. Complaints are registered before a notary and are obligatorily subject to review. Upon initial review, the board may solicit clarifications in writing, following which the board will determine either that the case and said explanations and justifications are grounded or that there are insufficient grounds to proceed with an investigation.^5^

Should the board determine that there are sufficient grounds to proceed, the complaint is referred to the disciplinary committee, which then names an investigator. Investigations proceed in accordance with the rules set forth in the Code of Medical Ethics. Once investigations have been instituted and completed, they are debated in plenary sessions and assigned to investigative fora, which may then find for or against the complainant, may order reconciliation between the parties or may order that a behavioral change contract for a given duration be signed.

Investigations judged to have insufficient grounds are dismissed; investigations judged to have sufficient grounds are automatically referred to the Case Disciplinary Committee, which names an evidence-gathering board for hearings involving the parties (complainant, defendant and witnesses) and then one board member as a rapporteur and reviewer for subsequent remittance of the professional-misconduct case to judgment.

Complaints may be lodged by individuals (patients, family members, neighbors or even doctors and other professionals), may be brought by the regional medical board (publicized in the media and originating from government agencies, the courts or medical associations) or may result from anonymous phone calls, written documentation or emails.^6^

According to 2007 data from CREMESP,^7^ the number of doctors against whom complaints were lodged in the state jumped from 2,023 in 2000 to 3,569 in 2006, which shows that proceedings brought against doctors in Brazil had reached critical levels over this six-year period, especially in the larger cities.

A review by the Regional Medical Council of the State of Goiás (Conselho Regional de Medicina de Goiás, CRM-GO) of complaints filed between 2000 and 2006^5^ showed that 62% of these complaints alleged professional incompetence and poor doctor-patient relationships. Seventy-three complaints corresponded to a mere four plastic surgeons, and one doctor was accused 49 times. The complaint was filed by an individual in 60% of the cases.

Between 2007 and 2009, the Regional Medical Council of the State of Minas Gerais (Conselho Regional de Medicina de Minas Gerais, CRM-MG) reviewed 411 complaints involving 518 doctors. Of these, 330 were absolved of the accusations, and 188 were disciplined with sanctions that ran the gamut from confidential warning, to confidential censure, public censure, 30-day suspension and license revocation.^8^

Silva et al.^9^ also showed that the Regional Medical Council of the State of Pará (Conselho Regional de Medicina do Pará, CRM-PA) registered a 15.34% increase in adjudicated medical board investigations but a 13.62% decrease in the number of medical board investigations opened between 2005 and 2007. Furthermore, despite this increase in the number of adjudicated investigations, there was a comparative 16.7% decrease in professional-misconduct cases reviewed between 2005 and 2007.

A further study^10^ surveyed the most common medical specialties cited in complaints that were reviewed by the CRM-PA and treated as professional-misconduct cases between 2006 and 2008. Among the 123 professional-misconduct cases that were reviewed over that period, obstetricians/gynecologists were cited most often (an average of 20.33% of the cases per year). In terms of classification, malpractice was the most frequent complaint, averaging 13 cases per year for each of the three years.

A study by Koeche, Cenci, Bortoluzzi and Bonamigo^11^ reviewed complaints filed before the Regional Medical Council of the State of Santa Catarina (Conselho Regional de Medicina de Santa Catarina, CREMESC) between January 2005 and December 2009.

They reviewed 468 professional-misconduct cases that were adjudicated due to violation of Article 29 of the 1998 Code of Medical Ethics. A total of 613 doctors were found to be in violation and appropriately judged; out of this number, 122 (19.9%) were found guilty of negligence, recklessness or professional malpractice, and 21 (17%) of these were convicted of medical malpractice. The majority (95.2%) were men; 35% had graduated from medical school 11-20 years earlier; 80.9% had been accused of more than one wrongdoing; and 71.4% were practicing as surgeons in the private healthcare system. General practitioners were the group most convicted (33.2%). The medical specialties with the greatest absolute numbers of convictions were obstetrics/gynecology (14.2%), anesthesiology (9.5%) and general surgery (9.5%).

The social impact of these medical malpractice complaints, which nearly always cause pain and suffering to patients and may involve poor doctor-patient relationships, is of great importance.^12^^,^^13^ The fact that a doctor is accused does not mean that she or he will be convicted, but medical professionals who are ordered to appear before a medical board for regional board investigations do worry, because they know that there may be irreversible consequences to their actions or errors.^14^

## OBJECTIVE

The objective of this paper was to classify CREMESP investigations among plastic surgeons that were reviewed between 2007 and 2016 and were treated as professional-misconduct cases.

## METHODS

This was a cross-sectional study in which 360 professional-misconduct cases were surveyed. These cases were reviewed and subjected to medical board investigation between 2007 and 2016. Out of the 360 cases reviewed, 8 (2.23%) were dismissed, 1 (0.27%) became an administrative lawsuit and 351 (97.50%) were treated as professional-misconduct cases per se. The final sample consisted of 351 cases.

This study was conducted via analysis of the cases in the CREMESP database following approval by the Santo Amaro University Research Ethics Committee (no. 2.338.983; on October 19, 2017) and by the president of CREMESP. Only complaints concerning the medical specialty of plastic surgery were reviewed, and the present authors did not have any access to the names of the doctors implicated therein. This study honored the principles of the Declaration of Helsinki and the Nuremberg Code through application of all ethics rules and the subsequent classification of complaints (pursuant to CFM Ruling 1785/2006). The professional-misconduct cases that were dismissed or that were converted into administrative lawsuits (cases that were suspended because the defendant developed a disabling disease that prohibited him/her from practicing medicine) were discarded from the sample.

The study reviewed medical cases pursuant to the protocol established by the present authors. The protocol consisted of questions concerning the nature of the complaints and the year in which the complaints were lodged. Excel 2007 was used to provide a quantitative analysis of the data based on types of variable. A descriptive statistical analysis was used to generate percentages from the data analyzed.

## RESULTS

A breakdown of the complaints over the period from 2007 to 2016 showed that complaints concerning malpractice (professional malpractice, recklessness or negligence) were the most common (28.43%), followed by complaints regarding medical advertising (24.19%) and poor doctor-patient relationships (10.39%).

**Figure 1.**
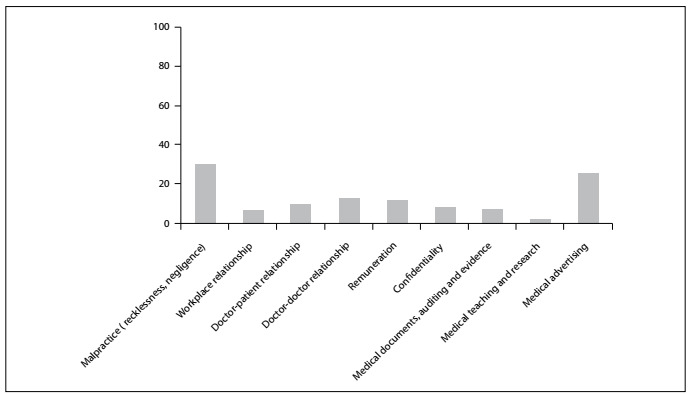
Percentage distribution of classification of complaints brought before CREMESP that were treated as professional-misconduct cases.

Figure 1 presents the classification of the complaints brought before CREMESP between 2007 and 2016 that were treated as professional-misconduct cases.

**Figure 2.**
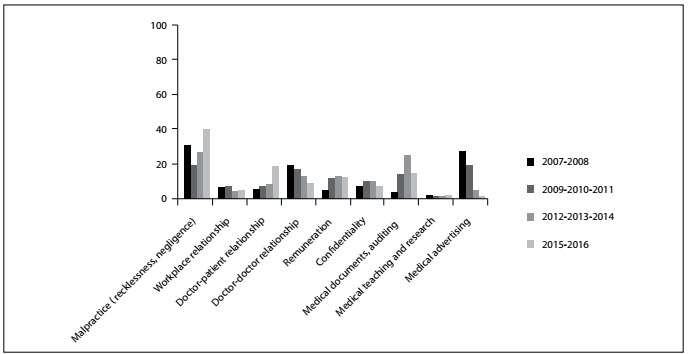
Percentage distribution according to the year, regarding the classification of complaints brought before CREMESP that were treated as professional-misconduct cases.

Figure 2 presents the classification of the complaints that were treated as professional-misconduct cases, according to the year in which they were brought before CREMESP. It can be seen the most common complaint for every year reviewed was malpractice, and that complaints of malpractice increased over the last two years reviewed (approximately 10%). Medical advertising complaints were alleged between 2009 and 2011, and complaints regarding medical documents, auditing and evidence between 2012 and 2014. Complaints concerning poor doctor-patient relationships increased by about 10% over the last two years reviewed.

## DISCUSSION

This paper analyzed 351 professional-misconduct cases that were reviewed between 2007 and 2016.

In terms of the nature of the complaints, medical malpractice was the most frequent complaint in every one of the nine years reviewed, accounting for a yearly average of 28.24% of the professional-misconduct cases. These complaints showed a 10% increase as a component of all complaints over the last two years reviewed (2015 and 2016). These statistics are in line with previous findings that have been published,^1^^,^^8^^,^^10^ thus corroborating that the most prevalent complaint filed with regional medical boards can be classified as medical malpractice (negligence, recklessness or professional malpractice).

Doctors are prohibited from engaging in medical practice that is harmful to patients and which can be characterized as professional malpractice, recklessness or negligence. This form of culpable practice may be adjudicated by the regional medical board as an ethical violation, or by the civil courts in cases of civil violation and award of damages, or by the criminal courts for prosecution of criminal behavior and application of subsequent penalties.

Negligence is evidenced by a lack of care and precaution when practicing medicine. It is characterized by inaction, indolence, inertia and passivity. It is effectively an act of omission.

Recklessness is the result of a doctor’s failure to anticipate the consequences of his/her acts or actions. Reckless doctors make unjustified, precipitated or imprudent decisions.

Lastly, professional malpractice occurs when the doctor demonstrates a lack of or inadequate technical medical knowledge or a lack of preparedness in medical practice. These harmful acts refer to professional conduct such as misdiagnosis, inadequate methods of treatment, improper post-operative care, wrongful drug prescriptions, anesthesia complications, surgical errors, wrongful early discharge and other problems that account for the most common departures from proper medical conduct. According to Cunha,^15^ despite technological advances in medical practice, including better diagnosis of a number of diseases and availability of new treatment options, doctors continue to make fundamental mistakes in the practice of medicine.

Complications are also a concern during surgery, considering that surgical procedures are more likely to result in adverse events and severe consequences that are more visible and more easily demonstrable.

Medical advertising was the second most common complaint over the nine years reviewed, although there were no complaints in this regard over the final two years studied. The CRMs state that there is no medical specialty officially recognized by the CFM in which the objective is esthetics. This means that the term “esthetic medicine” may not be used in a doctor’s or clinic’s advertising or marketing materials, since this creates a false sense that there is a discrete medical specialty known as “esthetic medicine.” This finding, that medical advertising was a common complaint, is backed by a 2017 study published by Shah et al.^16^

Esthetic procedures are practiced in a number of medical fields. The fact that there is a high demand for such procedures, given the current cultural and social standards of beauty and, consequently, that these procedures are financially lucrative, supports the findings of the present study.

However, doctors must not be influenced by perceived advantage, awards/prizes, increased clientele, financial gains, etc. Profiting from medicine through commercializing medicine constitutes anti-ethical behavior. Similarly, doctors are prohibited from appearing in commercial advertisements of any sort regarding their profession.

Furthermore, no doctor, regardless of medical specialty, may guarantee the result of a given treatment. Doctors must clearly inform the patient of the benefits and risks of any procedures. Publications in which a doctor advertises simple, fast, fully effective treatment are grounds for legal actions that hold him/her liable for the results. The promise of specific results puts doctors in a delicate spot since all procedures are subject to emergencies or unforeseen circumstances. Advertising should disclose information that is scientifically accurate and accepted as good medical practice. Doctors must always act in accordance with the law and ethical standards.

Complaints regarding poor doctor-patient relationships also increased by 10% over the final two years of the study period. The government has recently made a stronger push for greater civic participation, with reinforcement of the consumer protection code (Código de Defesa do Consumidor, CDC) and consumer protection agencies (Proteção ao consumidor, PROCON). It has fostered citizens’ awareness of their rights and protections to ensure that their needs as consumers are met, their dignity, health and safety are respected, and their economic interests are respected.

Introduction of the CDC was bold and innovative. It utterly reversed the status quo, meaning that consumers can now cite evidence of damage caused to them by vendors/practitioners. Doctors are considered to be service providers under the scope of the CDC, and the doctor-patient relationship may be referenced within the CDC more on account of inertia that on account of technical and legal grounds. Furthermore, the age-old doctor-patient relationship should not be conflated with the service provider-consumer relationship. It is also clear that unforeseen circumstances and increased workloads lead to a greater likelihood of malpractice suits. Nonetheless, poor relationships between doctors and their patients result in lawsuits that would otherwise be avoidable, were doctors simply to show better bedside manner.

## CONCLUSION

Among the professional-misconduct cases reviewed between 2007 and 2016 that were included in this study, those classified as malpractice (negligence, recklessness and professional malpractice) occurred most often (28.43%).

It was clear from the data that CREMESP has dealt with the issue of plastic-surgery complaints effectively and efficiently. Fluctuations and increases in the numbers of complaints over the nine-year period were significant. Advances in case proceedings ensured acceptable resolution rates. The severity of allegations brought before the board was addressed and penalized in a manner that was commensurate with what has been reported from other CRMs throughout Brazil.

It is important for doctors to keep in mind the meaning of the doctor-patient relationship, as essentially a more humanistic way of practicing medicine that respects patients and recognizes their dignity. Better doctor-patient relationships prevent complaints of medical malpractice and avoid a number of inconveniences and problems.

Greater investments in medical training are needed: investments that foster ongoing reflection and review of the ethical and humanistic precepts that shape humankind’s attitudes as social beings in familiar, affective, professional and political relationships, of both individual and collective nature. The aim therein should be to better incorporate awareness of biological, social and psychological elements into medical training and awareness of the full extent of the doctor-patient relationship, which is the cornerstone of medical practice.
